# The 2025 Westlake Autumn Symposium for AI Proteomics and Virtual Cell

**DOI:** 10.1093/gpbjnl/qzag022

**Published:** 2026-03-07

**Authors:** Rui Sun (孙瑞), Ruedi Aebersold, Qi Xiao (肖琦), Matthias Mann, Yan Zhou (周岩), Albert J R Heck, Yingrui Wang (王瑛睿), Uwe Völker, Xue Cai (蔡雪), Charles Boone, Zizhuo Zhou (周梓卓), Ming Li (李明), Zhen Dong (董振), Brenda Andrews, Shuaiyao Wang (王帅尧), Joseph Schacherer, Connie R Jimenez, Bernd Wollscheid, Liang Yue (岳靓), Ben C Collins, Heng Jiang (蒋恒), Dong Wang (王栋), Liujia Qian (钱鎏佳), Jia-Xing Yue (岳家兴), Yingying Sun (孙莹莹), Edouard Nice, Xiaofan Zhang (张晓帆), Yasset Perez-Riverol, Jun A (阿俊), Chris Sander, Henning Hermjakob, Wout Bittremieux, Wilson Wen Bin Goh, Ge Gao (高歌), Peijie Zhou (周沛劼), Siqi Sun (孙思琦), Han Wen (温翰), Ziyan Xu (许梓妍), Tiannan Guo (郭天南)

**Affiliations:** Affiliated Hangzhou First People’s Hospital, State Key Laboratory of Medical Proteomics, School of Medicine, Westlake University, Hangzhou 310000, China; Westlake Center for Intelligent Proteomics, Westlake Laboratory of Life Sciences and Biomedicine, Hangzhou 310000, China; Department of Biology, Institute of Molecular Systems Biology, ETH Zurich, 8093 Zurich, Switzerland; Affiliated Hangzhou First People’s Hospital, State Key Laboratory of Medical Proteomics, School of Medicine, Westlake University, Hangzhou 310000, China; Westlake Center for Intelligent Proteomics, Westlake Laboratory of Life Sciences and Biomedicine, Hangzhou 310000, China; Department of Proteomics and Signal Transduction, Max Planck Institute of Biochemistry, 82152 Martinsried, Germany; Affiliated Hangzhou First People’s Hospital, State Key Laboratory of Medical Proteomics, School of Medicine, Westlake University, Hangzhou 310000, China; Westlake Center for Intelligent Proteomics, Westlake Laboratory of Life Sciences and Biomedicine, Hangzhou 310000, China; Biomolecular Mass Spectrometry and Proteomics, Bijvoet Center for Biomolecular Research and Utrecht Institute for Pharmaceutical Sciences, Utrecht University, Utrecht, 3584 CH, The Netherlands; Affiliated Hangzhou First People’s Hospital, State Key Laboratory of Medical Proteomics, School of Medicine, Westlake University, Hangzhou 310000, China; Westlake Center for Intelligent Proteomics, Westlake Laboratory of Life Sciences and Biomedicine, Hangzhou 310000, China; Department of Functional Genomics, Interfaculty Institute of Genetics and Functional Genomics, University Medicine Greifswald, 17489 Greifswald, Germany; German Centre for Cardiovascular Research (DZHK), partner site Greifswald, 17489 Greifswald, Germany; Affiliated Hangzhou First People’s Hospital, State Key Laboratory of Medical Proteomics, School of Medicine, Westlake University, Hangzhou 310000, China; Westlake Center for Intelligent Proteomics, Westlake Laboratory of Life Sciences and Biomedicine, Hangzhou 310000, China; The Donnelly Centre, University of Toronto, Toronto, ON M5S 3E1, Canada; Department of Molecular Genetics, University of Toronto, Toronto, ON M5S 3E1, Canada; Affiliated Hangzhou First People’s Hospital, State Key Laboratory of Medical Proteomics, School of Medicine, Westlake University, Hangzhou 310000, China; Westlake Center for Intelligent Proteomics, Westlake Laboratory of Life Sciences and Biomedicine, Hangzhou 310000, China; Central China Institute of Artificial Intelligence, Zhengzhou 450000, China; Bioinformatics Solutions Inc., Waterloo, ON N2L 3G1, Canada; Cheriton School of Computer Science, University of Waterloo, Waterloo, ON N2L 3G1, Canada; Affiliated Hangzhou First People’s Hospital, State Key Laboratory of Medical Proteomics, School of Medicine, Westlake University, Hangzhou 310000, China; Westlake Center for Intelligent Proteomics, Westlake Laboratory of Life Sciences and Biomedicine, Hangzhou 310000, China; The Donnelly Centre, University of Toronto, Toronto, ON M5S 3E1, Canada; Department of Molecular Genetics, University of Toronto, Toronto, ON M5S 3E1, Canada; Affiliated Hangzhou First People’s Hospital, State Key Laboratory of Medical Proteomics, School of Medicine, Westlake University, Hangzhou 310000, China; Westlake Center for Intelligent Proteomics, Westlake Laboratory of Life Sciences and Biomedicine, Hangzhou 310000, China; Université de Strasbourg, National Center for Scientific Research, Molecular Genetics, Genomics, Microbiology, Unité Mixte de Recherche 7156, 67000 Strasbourg, France; OncoProteomics Laboratory, Department of Medical Oncology, Amsterdam University Medical Center, location Vrije Universiteit Amsterdam, Amsterdam, 1081 HV, The Netherlands; Cancer Center Amsterdam, Amsterdam, 1081 HV, The Netherlands; Department of Biology, Institute of Molecular Systems Biology, ETH Zurich, 8093 Zurich, Switzerland; Affiliated Hangzhou First People’s Hospital, State Key Laboratory of Medical Proteomics, School of Medicine, Westlake University, Hangzhou 310000, China; Westlake Center for Intelligent Proteomics, Westlake Laboratory of Life Sciences and Biomedicine, Hangzhou 310000, China; School of Biological Sciences, Queen’s University of Belfast, Belfast, BT9 5AG, UK; Affiliated Hangzhou First People’s Hospital, State Key Laboratory of Medical Proteomics, School of Medicine, Westlake University, Hangzhou 310000, China; Westlake Center for Intelligent Proteomics, Westlake Laboratory of Life Sciences and Biomedicine, Hangzhou 310000, China; Chengdu University of Traditional Chinese Medicine, Chengdu 611137, China; Affiliated Hangzhou First People’s Hospital, State Key Laboratory of Medical Proteomics, School of Medicine, Westlake University, Hangzhou 310000, China; Westlake Center for Intelligent Proteomics, Westlake Laboratory of Life Sciences and Biomedicine, Hangzhou 310000, China; State Key Laboratory of Oncology in South China, Guangdong Key Laboratory of Nasopharyngeal Carcinoma Diagnosis and Therapy, Guangdong Provincial Clinical Research Center for Cancer, Sun Yat-sen University Cancer Center, Guangzhou 510060, China; Affiliated Hangzhou First People’s Hospital, State Key Laboratory of Medical Proteomics, School of Medicine, Westlake University, Hangzhou 310000, China; Westlake Center for Intelligent Proteomics, Westlake Laboratory of Life Sciences and Biomedicine, Hangzhou 310000, China; Department of Biochemistry and Molecular Biology, Monash University, Clayton, VIC 3800, Australia; Affiliated Hangzhou First People’s Hospital, State Key Laboratory of Medical Proteomics, School of Medicine, Westlake University, Hangzhou 310000, China; Westlake Center for Intelligent Proteomics, Westlake Laboratory of Life Sciences and Biomedicine, Hangzhou 310000, China; European Molecular Biology Laboratory, European Bioinformatics Institute (EMBL-EBI), Wellcome Trust Genome Campus, Cambridge, CB10 1SD, UK; Affiliated Hangzhou First People’s Hospital, State Key Laboratory of Medical Proteomics, School of Medicine, Westlake University, Hangzhou 310000, China; Westlake Center for Intelligent Proteomics, Westlake Laboratory of Life Sciences and Biomedicine, Hangzhou 310000, China; Department of Systems Biology, Harvard Medical School, Boston, MA 02115, USA; Broad Institute of Harvard and Massachusetts Institute of Technology, Boston, MA 02115, USA; Ludwig Center at Harvard, Boston, MA 02115, USA; European Molecular Biology Laboratory, European Bioinformatics Institute (EMBL-EBI), Wellcome Trust Genome Campus, Cambridge, CB10 1SD, UK; Department of Computer Science, University of Antwerp, 2020 Antwerp, Belgium; Lee Kong Chian School of Medicine, Nanyang Technological University, Singapore 639798, Singapore; State Key Laboratory of Protein and Plant Gene Research, School of Life Sciences, Biomedical Pioneering Innovative Center (BIOPIC) & Beijing Advanced Innovation Center for Genomics (ICG), Center for Bioinformatics (CBI), Peking University, Beijing 100871, China; Academy for Advanced Interdisciplinary Studies, Peking University, Beijing 100871, China; AI for Science Institute, Beijing 100080, China; Center for Machine Learning Research, Peking University, Beijing 100871, China; Research Institute of Intelligent Complex Systems, Fudan University, Shanghai 200438, China; AI for Science Institute, Beijing 100080, China; DP Technology Co., Ltd., Beijing 100080, China; Beijing Advanced Center of RNA Biology (BEACON), Peking University, Beijing 100871, China; State Key Laboratory of Medical Proteomics, Beijing 100850, China; Affiliated Hangzhou First People’s Hospital, State Key Laboratory of Medical Proteomics, School of Medicine, Westlake University, Hangzhou 310000, China; Westlake Center for Intelligent Proteomics, Westlake Laboratory of Life Sciences and Biomedicine, Hangzhou 310000, China; Affiliated Hangzhou First People’s Hospital, State Key Laboratory of Medical Proteomics, School of Medicine, Westlake University, Hangzhou 310000, China; Westlake Center for Intelligent Proteomics, Westlake Laboratory of Life Sciences and Biomedicine, Hangzhou 310000, China

## Introduction

Artificial intelligence (AI) is transforming life science research by enabling the analysis of complex, high-dimensional biological data. In mass spectrometry (MS)-based proteomics, AI improves peptide and protein identification and quantification, facilitates systematic characterization of protein–protein interactions and complexes, advances spatiotemporal protein analysis and multi-omics data integration, and ultimately contributes to the construction of AI virtual cell (AIVC). By capturing the underlying native dynamics of cellular states, AIVC has the potential to predict disease progression, accelerate drug discovery, and guide precision medicine [1–3]. Advancing this ambitious vision requires the collective effort of the scientific community. To this end, leading experts were invited to contribute to discussions on AI proteomics and the development of AIVC.

The Fourth Westlake Symposium on AI Proteomics and Virtual Cell convened on October 8–9, 2025, at Westlake University in Hangzhou, China, fostering interdisciplinary dialogue essential for constructing mechanistic cellular models. Organized by Tiannan Guo, the symposium united experimental and computational leaders to address complementary facets of AI proteomics and virtual cell. Ruedi Aebersold, Matthias Mann, Albert J.R. Heck, and Uwe Völker underscored that advances in proteomics technologies and AI-driven interpretation of MS data are revolutionizing the depth, precision, and biological relevance of protein measurements. Charles Boone, Brenda Andrews, Joseph Schacherer, and Jia-Xing Yue presented yeast systems biology as a blueprint for network-level cellular understanding. Bernd Wollscheid, Ben C. Collins, Dong Wang, Connie R. Jimenez, and Edouard Nice demonstrated translational applications bridging proteomics discoveries to precision therapeutic strategies. Yasset Perez-Riverol, Ming Li, Chris Sander, Henning Hermjakob, Wout Bittremieux, Wilson Wen Bin Goh, Peijie Zhou, Siqi Sun, and Han Wen contributed computational perspectives on data integration, machine learning architectures, and foundation models for biological prediction.

In this report, we provide a concise overview of the presentations and summarize key highlights from each talk. We conclude with reflections on the symposium in fostering international collaboration and advancing the vision of building the AI-powered virtual cell.

## Topic

### Technological development of AI proteomics toward virtual cells

This session showcased how recent technological breakthroughs — from AI-driven spectral interpretation and high-throughput platforms to spatiotemporal subcellular profiling and direct immune repertoire sequencing — are generating the comprehensive, multi-dimensional datasets essential for training generative models capable of simulating cellular responses and informing precision therapeutic interventions.

Tiannan Guo provided an overview of recent progress in subcellular spatiotemporal proteomics and its potential application in constructing virtual cells and the Westlake AI Virtual Cell Yeast (WAY) project. He proposed three essential components for constructing virtual cells, including elements, organization, and dynamics. Further, he introduced three essential data pillars for constructing the AIVC framework: prior knowledge, static architecture, and dynamic states supported by closed-loop active learning. Building a virtual cell still requires generative AI technologies. Modern generative AI can transform a blurry, fragmented image into a clear and complete one, or even turn a static picture into a dynamic video. Similarly, AIVC aims to reconstruct the full dynamic behaviors of a cell from incomplete omics data. Tiannan’s team has accumulated large-scale spatiotemporal proteomics datasets, achieving subcellular proteomics with tissue expansion technology. The filter-aided expansion proteomics (FAXP) method enables the quantitative identification of about 2500 proteins from a single mouse liver cell nucleus. The team has also generated large-scale, time-resolved perturbation proteomics datasets, and constructed a proteome foundation model called ProteinTalks (v1.0) for drug response prediction. Together with multidisciplinary colleagues, they propose a working plan for constructing virtual yeast.

In the following presentation, Ruedi Aebersold from ETH Zurich described ongoing efforts to digitize proteomic spaces across tissues and individuals to advance mechanistic understanding and disease prediction. Motivated by high clinical trial failure rates that are caused, at least in part, by a lack of deep understanding of the biology of the respective drug target and the contextual changes caused by drug–target engagement, he argues that biology needs models grounded in molecular mechanisms, not just pattern recognition. As a stepping stone, an AI virtual yeast cell is proposed. The well-characterized genome, well-understood and moderately complex proteome, and experimental amenability of yeast support dense, multi-PROTeomic measurements spanning proteins, proteoforms, post-translational modifications (PTMs), interactions, structure, activity, and RNA–protein contacts. Ruedi framed the cell as a complex adaptable system: diverse agents adapt their states, interact locally, and generate emergent behaviors. Case studies show coordinated proteomic and phosphoproteomic waves across meiosis — hundreds of proteins and multiple kinases changing states — and variant mapping across the transcriptome, proteome, and phosphoproteome, where correlates of physiological traits increasingly arise nearer to phenotype along the central-dogma axis. These implications highlight the need to systematically perturb cells and measure hallmarks on the same samples over time, centralize or tightly coordinate data collection, assess which data types most effectively support mechanistic modeling, and iteratively refine the models based on these insights. With its relative simplicity and scale, yeast is positioned as an AIVC test bed, ultimately advancing our understanding of the principles governing functional cellular states as captured by AI virtual cells, and enabling precise disease prediction and prevention in humans.

Cutting-edge developments in proteomics technology and biomedical applications from Matthias Mann’s group at the Max Planck Institute of Biochemistry exemplify how AI is reshaping molecular discovery. Their toolkit includes AlphaPeptDeep for peptide prediction, AlphaDIA and AlphaNovo for direct deep learning-based spectral decoding, and large-scale model training pipelines that collectively enable the analysis of up to 500 proteomes per day using the Evosep–Astral platform. This unprecedented throughput expands the scope of affinity-enrichment applications, ranging from yeast protein interactome and virus–human interaction mapping to proteome-wide drug screening using thermal shift and peptide-centric local stability assay (PELSA) chemoproteomics approaches. The same framework also supports large-scale plasma proteomics, facilitating studies of fatty liver disease, pediatric cohorts, and mother–infant multi-omics at an annual throughput exceeding 50,000 samples. By combining high-throughput proteomics with AI-driven analysis, Mann’s team developed the Adaptive Diagnostic Architecture for Personalized Testing by MS (ADAPT-MS) — a machine learning framework that enables direct diagnostic and prognostic interpretation of discovery-mode proteomics at the level of individual samples. In spatial proteomics, the group has integrated multiplexed antibody staining into the Deep Visual Proteomics (DVP) workflow, enabling organ- and cell-type-resolved analyses across brain, lymph node, lung, liver, kidney, heart, skin, and ovary tissues. These studies reveal organ-specific heterogeneity, disease-associated spatial proteomic signatures, and candidate therapeutic targets. Collectively, these advances illustrate the frontier of AI-integrated, high-throughput proteomics — bridging molecular discovery, virtual cell modeling, and precision medicine.

Recent advances from Albert J.R. Heck’s group at Utrecht University (the Netherlands) have opened new frontiers in profiling often-overlooked blood molecules, as presented in a lecture entitled “*What do we know about (your) antibodies?*”. He described MS-based antibody repertoire profiling and *de novo* sequencing of endogenous antibodies. This technology enables the direct decoding of the unique antibody repertoire generated by individuals in response to pathogens, facilitating the identification of optimal candidate molecules for development into biotherapeutics. Immunoglobulins are among the most abundant proteins in our blood and throughout the body. They play a key role in our humoral immune system, protecting us against microbial infections. By capturing blood-derived IgG1 and utilizing innovative techniques such as electron-activated dissociation coupled with collision-induced dissociation fragmentation (EaciD) and novel high-temperature active (HTA) proteases, the method achieves high-quality and high-coverage antibody sequence resolution. This approach has been successfully applied to directly discover potent IgGs against emerging variants (*e.g.*, the SARS-CoV-2 Omicron sublineage, JN.1) from the plasma of COVID-19 patients and vaccinated donors. These findings establish the approach as a transformative strategy for rapidly discovering highly effective human antibodies from patient samples — a vision actively pursued through Abvion, a company co-founded by Professor Heck.

In the domain of population-based omics research, Uwe Völker from the University Medicine Greifswald provided a systematic overview of the strengths, advances, and challenges. He outlined how the field benefits from well-defined standardized workflows, strong international cooperative networks, and increasingly cost-effective high-throughput technologies, which serve as key drivers for progress. Using large cohorts like the Study of Health in Pomerania (SHIP) and the UK Biobank as examples, he illustrated how such studies, by integrating multi-omics data, can provide profound insights into the biological basis of health and disease. He highlighted recent breakthroughs in plasma proteomics, emphasizing that the significantly increased throughput and coverage now enable large-scale cohort analyses and identification of numerous protein–genetic–health associations. He cited dual-laboratory studies between his lab and that of Albert J.R. Heck to demonstrate that robust reproducibility and comparability can be achieved across different sites with proper normalization, despite methodological variations. However, he also candidly addressed ongoing challenges, including complexities in data sharing and protection, pre-analytical sample handling variations, and a lack of universal reference standards. Concluding with a forward-looking perspective, he stressed that in the era of big science, enhanced international collaboration via platforms like the Human Proteome Organization (HUPO), coupled with the establishment of standardized frameworks and reference materials, will be crucial for integrating diverse datasets and ultimately advancing toward early disease detection and precision medicine.

Collectively, the discussions underscored a paradigm shift in proteomics — from protein measurement to comprehensive, data-driven understanding of biological systems. Advances in spatial, temporal, and perturbation proteomics, alongside large-scale population studies and antibody repertoire decoding, are generating multidimensional datasets that capture the dynamic and diverse nature of biology. With the integration of generative AI and computational modeling, proteomics is evolving into a predictive and mechanistic science. This convergence is redefining how molecular data are measured, interpreted, and applied, bridging discovery research and translational medicine toward a truly systems-level view of life. While experimental proteomics technologies lay the crucial data foundation for AIVC, translating these massive datasets into predictive cellular models demands sophisticated computational frameworks and AI methodologies.

### Perspective from AI scientists

The AI session brought together diverse perspectives on how AI can accelerate the construction of the AIVC. Discussions spanned from establishing standardized data foundations to developing innovative modeling strategies that integrate causality, feature representation, and multimodal biological information.

Publicly available large-scale datasets serve as essential platforms for bioinformatics analysis. Yasset Perez-Riverol at EMBL-EBI provided a comprehensive overview of the quantms platform (https://quantms.org), outlining its design principles and technological innovations aimed at bridging proteomics and AI. He emphasized the current challenges in the field, including inconsistent metadata and data formats, variability in processing workflows, and limited reusability of datasets for machine learning applications. To address these issues, quantms offers a fully integrated ecosystem featuring standardized data structures, modular algorithms, and automated processing pipelines to convert raw MS data into high-quality, AI-compatible formats. He detailed improvements across key stages such as data cleaning, retention time alignment, feature extraction, and consistency control in quantification. A particular focus was placed on the role of unified data formats in enhancing dataset interoperability and reproducibility across studies. He concluded by highlighting quantms’ seamless compatibility with downstream machine learning frameworks, positioning it as a powerful and scalable platform for AI-driven proteomics research.

Progress from Henning Hermjakob’s team at EMBL-EBI exemplifies how pathway informatics is evolving toward intelligent, interactive systems. The latest Reactome (v4) introduces dynamic pathway visualization, real-time graph reconstruction, and an AI-powered chat assistant that allows users to explore signaling cascades through natural language queries. The new version supports automatic tracing of upstream and downstream signaling pathways, significantly improving the accessibility and interpretability of pathway data. A live demonstration showed how users can interact with Reactome using natural language queries, enabling rapid identification of key molecular events and functional modules. These advancements not only reinforce Reactome’s position as a high-quality open-access database but also establish it as a powerful intelligent platform for systems biology research.

AI-powered MS data mining involves using advanced AI techniques to analyze and interpret complex MS data, enabling more accurate identification of proteins, peptides, and other biomolecules for various applications in proteomics and biomedical research. Wout Bittremieux from the University of Antwerp introduced a community-driven benchmarking platform for *de novo* peptide sequencing tools, comprising 29 datasets with 24 million spectra, of which 6.4 million spectra were ground truth annotated using a combination of three different sequence database search engines. These data cover a wide range of organisms (human, animals, plants, and microbes), digestion enzymes, and MS instruments, with applications in immunopeptidomics, metaproteomics, single-cell proteomics, antibody sequencing, and PTM analysis. The platform further integrates 18 *de novo* sequencing tools, each with standardized runtime environments and parameters, allowing systematic comparison between predicted peptide spectrum matches (PSMs) and ground-truth PSMs to compute peptide- and amino acid-level performance metrics. Importantly, the *de novo* tools can be continuously updated, making this an evolving benchmarking resource. Overall, this platform provides a comprehensive and dynamic evaluation of AI-based *de novo* peptide sequencing tools, making a significant contribution to advancing this field.

Adding to this momentum, Ming Li from the University of Waterloo and the Central China Institute of Artificial Intelligence delivered a talk entitled “*Deciphering the Human Immunopeptidome by AI*”. He introduced an AI- and MS-based strategy to comprehensively decode the human immunopeptidome. Ming highlighted that immune imbalance underlies both cancer and autoimmune diseases, and that building a complete immunopeptidome database is key to advancing personalized immunotherapy. His team has developed innovative solutions for challenges in formalin-fixed, paraffin-embedded (FFPE) sample de-crosslinking, identification of noncanonical peptides and PTMs, AI-based peptide database search, and T cell receptor–peptide–major histocompatibility complex (TCR–pMHC) binding prediction using reinforcement learning. He concluded by outlining plans to systematically profile peptides across autoimmune and cancer tissues and to establish a public immunopeptidome database, paving the way for AI-driven precision immunology.

Different AI experts proposed distinct approaches for building the AIVC. For single molecules, Siqi Sun from Fudan University introduced IntelliFold, a controllable foundational model that has achieved breakthroughs in both general and specialized biomolecular structure prediction. He began by reviewing the technological advancements in *de novo* peptide sequencing and protein structure prediction, while also highlighting the challenges that current models face in complex scenarios, such as antibody–antigen and protein–nucleic acid complexes, under the FoldBench benchmark. The core strengths of IntelliFold were then elaborated: it supports high-accuracy prediction across multiple modalities, including proteins and nucleic acids; delivers ultra-fast inference with low memory consumption; and enables precise modeling of key structural features, such as allosteric sites, through customizable constraints.

For more complex biological systems, four AI experts — Chris Sander, Ge Gao, Peijie Zhou, and Han Wen — proposed distinct AI-based approaches for constructing the AIVC.

At the leading edge of conceptual development, the vision of “perturbation biology”, articulated by Chris Sander of Harvard Medical School, seeks to construct computable and predictive models of cellular systems through systematic perturbations and high-dimensional molecular and phenotypic response measurements. He highlighted challenges such as moving beyond single-protein measurements using powerful protein MS, capturing temporal state changes, and using domain knowledge to guide AI processes. His work highlights practical achievements through AI-powered protein design (*e.g.*, using EVcouplings to engineer improved plastic-degrading enzymes) and through drug target discovery that focuses on AI-inferred cellular processes and perturbation combinations. Furthermore, he reported advances in machine learning for disease prevention, exemplified by AI models such as CancerRiskNet to stratify pancreatic cancer risk from clinical records for the goal of early cancer detection and early intervention. The goal is to build executable, multi-scale models — from cells to tissues and organs — that enable quantitative prediction of therapeutic perturbations and advance personalized medicine to extend healthy life spans.

Complementing this vision, the framework proposed by Ge Gao from Peking University focuses on building a “causality-oriented cell *in silico*” that moves beyond canonical association-based prediction toward mechanistic understanding and rational design. He highlighted that while multi-omics technologies have revealed cellular heterogeneity and complexity of gene regulatory networks, deciphering their underlying causal hierarchy remains a major challenge. To bridge this gap, his team proposes integrating massive omics data with cutting-edge AI and machine learning to develop a suite of “causality-oriented generative models”. Combining data-driven and knowledge-based principles, his team aims to create a “Regulatory Language Model” for complex eukaryotic cells, which would ultimately enable the *in silico* simulation and rational design for cell transcriptomic reprogramming within a unified computational framework.

Extending these ideas toward model architecture and learning dynamics, Peijie Zhou from Peking University emphasized the future of AIVC, challenging the current reliance on foundation models inspired by large language models. He argued that three key principles should guide AIVC development. First, foundation models using semantic vectors may not be the complete solution, as recent benchmarking shows that they often face challenges in outperforming traditional methods and struggle with generative tasks, such as perturbation prediction. Second, AIVC should incorporate biological priors rather than being purely black-box models — explicitly modeling processes like cell cycle, fitness changes, and growth dynamics improves accuracy. For instance, for the spatiotemporal generation task, Peijie advocated for “biological transport” rather than just optimal transport, using mechanistic models combined with neural networks to balance interpretability and predictive power. Third, AIVC represents a paradigm shift toward active learning, where models guide experimental design rather than passively analyzing data. He proposed that diffusion models might be a promising alternative to language model approaches for data-constrained scenarios of the generation task, and introduced simulation-free methods, like flow matching for efficient generative modeling. The ultimate vision of AIVC is to create closed-loop systems combining generative capabilities, mechanistic understanding, and active experimental guidance.

At the implementation and systems-integration levels, Han Wen from Peking University introduced Uni-RNA, a large-scale pre-trained model for nucleic acid sequences recently developed by his team. This model has demonstrated excellent performance in tasks such as RNA structure prediction and functional annotation, significantly enhancing modeling capabilities at the nucleic acid level within multi-omics research. He further explored the application potential of large foundation models and neural dynamic systems in life science omics research, particularly in modeling complex biological systems and enabling virtual experimental simulations. Building upon these advances, Han proposed that the concept of the AIVC could be realized through the integration of multimodal and dynamical foundation models. By incorporating heterogeneous biological data sources — including transcriptomics, proteomics, spatial omics, and microscopy imaging — AIVC aims to construct a unified, temporally-aware, and context-sensitive representation of cellular states. He emphasized that such a framework would overcome the limitations of traditional static cell modeling, enabling researchers to realistically simulate cellular dynamics under various perturbations and to establish a more intelligent computational platform for mechanistic discovery, drug screening, and disease modeling.

During the session on AI, it is well accepted that establishing data foundations is necessary to build a common standard for AI training. However, when it comes to building the AI itself, there are a myriad of perspectives, ranging from causality modeling to feature engineering to compressing large data vectors for effective representation and integration with state-of-the-art foundation models, bringing in additional layers of critical information such as gene expression.

### Biomedicine applications

With rapid advances in proteomics technologies and AI, biology is entering an era where complex cellular systems can be digitally reconstructed. Integrating large-scale proteomic, genomic, and phenotypic datasets with AI enables modeling of molecular interactions, cellular organization, and dynamic responses, laying the foundation for building AIVCs that can bridge experimental biology and predictive medicine.

The development of AIVCs, initially focused on a virtual yeast prototype using *Saccharomyces cerevisiae*, was the subject of several presentations. Charles Boone from the University of Toronto demonstrated the profound conservation of genetic interaction (GI) networks from yeast to human cells. He leveraged high-throughput screening to construct global GI maps in both yeast (CellMap) and a human cell line (HAP1). This comparative analysis revealed that the fundamental architectural principles, such as the functional clustering of genes, are highly conserved. The team further provided direct evidence for the conservation of specific interaction profiles for orthologous genes (*e.g.*, yeast *Ecm9* and human *PTAR1*), validating the use of yeast as a model. By integrating these networks with other large-scale datasets using their deep learning framework (BIONIC), their work provides a powerful, scalable strategy to elucidate human gene function and genetic basis of disease.

Complementing these network-level insights, Brenda Andrews, also from the University of Toronto, introduced a high-throughput “phenomics” platform that combines Synthetic Genetic Array (SGA) technology with automated single-cell imaging in yeast to systematically study cellular organization. By perturbing essential genes, the study revealed that cellular collapse is not stochastic but follows an exponential decay pattern propagated by rapid cascade effects. The trajectory of this collapse is highly specific to the initial bioprocess perturbed; for instance, inhibiting vesicle trafficking causes an acute and widespread loss of organization. Crucially, the degree and speed of this structural loss are highly predictive of cell death. The study extends this model to natural cellular aging, showing a similar exponential collapse initiated by early defects, often in mitochondrial function, toward the end of a cell’s lifespan.

Expanding the foundation for genotype–phenotype mapping, Joseph Schacherer and his lab from the University of Strasbourg generated near telomere-to-telomere genome assemblies for 1086 *Saccharomyces cerevisiae* isolates, enabling an unprecedented exploration of the genotype–phenotype relationship. This resource includes a highly complete atlas of 44,804 structural variants (SVs) alongside single nucleotide polymorphisms (SNPs) and small insertions and deletions (indels), and integrates these with 8391 molecular and organismal phenotypes. Incorporating SVs and indels increased narrow-sense heritability estimates by an average of 14.3% compared with SNP-only. The study shows that SVs are more frequently associated with traits than SNPs or indels, display markedly higher pleiotropy, and disproportionately underlie quantitative trait locus (QTL) hotspots. Notably, organismal traits exhibit a more polygenic architecture with many small-effect variants, and are more strongly enriched for SV-QTL than molecular traits. Overall, this work demonstrates the central role of SVs in shaping complex phenotypes and provides a nearly complete variant catalog for addressing missing heritability in eukaryotes.

Telomeres play a crucial role in maintaining genome integrity, with important implications in both aging and cancer. However, existing sequencing approaches have inherent limitations in efficiently and accurately characterizing telomeric regions. Jia-Xing Yue from Sun Yat-sen University Cancer Center presented a universal telomere-targeted sequencing method and demonstrated its robust performance in multiple study systems, including humans, yeast, and mice. Applying this method to yeast genetic perturbation and mouse aging studies revealed significant changes in telomere length in both contexts. Furthermore, in cancer cell lines, the approach uncovered pronounced genomic instability, manifested in neotelomere dynamics. Finally, the association between telomere dynamics and cancer drug resistance, as well as the efficacy of applying a telomerase inhibitor to reverse cancer drug resistance, was shown, highlighting the therapeutic potential of telomere-targeted interventions.

Drug discovery will benefit from AIVC, and the next three presentations focused on proteomic technologies supporting drug discovery. Bernd Wollscheid from ETH Zurich explored how machine learning and AI could be utilized to decode the functional architecture of the surfaceome — the dynamic community of proteins on the cell surface. The presentation highlighted that the architecture of the surfaceome remains unexplored, and introduced technologies like Cell Surface Capture (CSC) and LUX-MS for mapping surface proteins and their nanoscale interactions. The machine learning tool SURFY helps predict surfaceome proteins, while LUX-MS enables light-controlled, time-resolved analysis of protein communities. Applications include profiling cancers (*e.g.*, lymphoma and lung cancer), studying neuronal development, and analyzing immunotherapy-induced synapses. The goal is to create a dynamic virtual “Google Street Map” of the surfaceome as a basis for the rational design of and development of new therapeutic strategies, supported by collaborative efforts and large-scale multi-omics data integration.

Parallel advances in chemoproteomics are expanding the boundaries of druggable biology. Ben C. Collins from Queen’s University of Belfast presented applications of chemoproteomics in drug discovery and opportunities for AI integration. He highlighted two emerging drug modalities addressing “undruggable” proteins: targeted protein degradation [proteolysis-targeting chimeras (PROTACs) or molecular glues] and covalent ligands. The Collins lab has developed high-throughput sample preparation and data acquisition workflows, enabling time- and concentration-resolved assessment of protein degradation. He demonstrated a dual global proteomics and activity-based protein profiling approach that simultaneously measures protein abundance and covalent ligand engagement in single experiments. Regarding AI applications, Collins discussed the potential of protein–ligand structure prediction models like AlphaFold3 and Boltz-2, proposing that chemoproteomics datasets could serve as valuable training data for affinity prediction models. Such datasets capture biologically relevant, context-dependent liganding events, potentially addressing limitations of current training data derived mainly from the Protein Data Bank. He suggested developing local machine learning models based on chemoproteomics data to drive virtual screening and prioritize compounds for experimental validation in specific biological contexts.

Complementing proteomic innovation, high-throughput transcriptomic profiling platforms have dramatically accelerated large-scale compound screening. Dong Wang from Chengdu University of Traditional Chinese Medicine presented the use of high-throughput transcriptomic technologies for drug discovery. He introduced HTS^2^ and the newly developed HiMAP-seq method, which enables profiling of thousands of genes across thousands of samples in a single run with high sensitivity, reproducibility, and minimal index misassignment. Using these tools, his team built the CIGS database, comprising ∼ 320 million gene expression events from over 13,000 compounds across two human cell lines. The resource was applied to discover novel Bromodomain protein 4 (BRD4) inhibitors (*e.g.*, ligustroflavone) and ferroptosis-resistant compounds (*e.g.*, 2,4-dihydroxypteridine), demonstrating its utility for uncovering mechanisms of unannotated molecules and traditional Chinese medicine ingredients. CIGS serves as a foundational dataset for AI-driven drug discovery and systems biology.

Meanwhile, advances in AI-enhanced proteomics pipelines are redefining how large-scale datasets are analyzed and interpreted. Connie R. Jimenez, Professor at the Translational OncoProteomics Laboratory of Amsterdam University Medical Center, together with computer scientist Thang Pham’s team, is dedicated to integrating MS-based proteomics with AI. Her presentation showcased how Transformer deep learning models can enhance data-independent acquisition MS (DIA-MS) phosphoproteomics data interpretation, including the development and optimization of retention time, MS/MS, and ion mobility prediction models. The team trained these models on large-scale datasets and released the open-source package aiproteomics. They also introduced the software package *iq* (v2.0) for large-scale protein, peptide, and site quantification. Their work enables precise analysis of cancer signaling pathways and offers new perspectives for personalized cancer therapy.

The convergence of AI and proteomics is also extending into psychiatry, where molecular signatures are poised to transform diagnosis and treatment. Wilson Wen Bin Goh from Nanyang Technological University presented a program on how proteomics can be used for mental health diagnosis and treatment planning by addressing the limitations of current symptom-based assessments, which are often subjective and influenced by cultural and clinical variability. He highlighted that proteomics enables the identification of molecular biomarkers underlying psychiatric disorders through high-resolution technologies such as the Astral mass spectrometer, and that coupling these data with AI — including deep learning and multimodal large language models — can reveal predictive patterns for early detection and therapeutic response. His talk proposed establishing Singapore’s first national-level transdiagnostic mental health proteome covering schizophrenia, bipolar disorder, depression, anxiety, and comorbidity cohorts, with use cases on predicting treatment resistance, improving psychosis risk prediction in Asian populations, and enhancing dataset readiness for AI integration. Together, these efforts seek to reimagine precision psychiatry and create a scalable, globally relevant framework for objective mental health diagnosis and prognosis.

Finally, Edouard Nice from Monash University summarized the rapid advancements in omics technologies, noting that the completion of the human genome and proteome is now over 90%. He highlighted the intense focus on AI technology in personalized medicine and its role in fostering interdisciplinary international collaboration. He outlined how AI will redefine pathology and the new responsibilities for pathologists. Furthermore, he discussed the personalized medicine market, the emerging directions in proteomics, and the potential hurdles encountered when using AI to achieve precision medicine.

Presentations showcased how multi-omics integration and AI-driven modeling advance the construction of virtual cells. From conserved genetic interaction networks and cellular organization dynamics in yeast to structural variation mapping, telomere profiling, and chemoproteomics-guided drug discovery, these studies demonstrated how proteome-centered datasets and machine learning approaches can capture biological complexity, guiding AIVC development and its translation into precision therapeutics.

## Conclusion

From enhancing protein quantification and spatial proteomics to developing predictive models for disease diagnostics and drug discovery, AI-powered tools are significantly improving our ability to understand complex biological systems. Moving forward, the continued evolution of AI models, along with large-scale multi-omics datasets, will be crucial in building more accurate, dynamic, and scalable models of cellular behaviors, offering promising avenues for precision medicine, disease prevention, and therapeutic development. Many participants have expressed their willingness to attend such events in the future to further advance the field.

## CRediT author statement

**Rui Sun:** Writing – original draft, Writing – review & editing. **Ruedi Aebersold:** Writing – review & editing. **Qi Xiao:** Writing – original draft. **Matthias Mann:** Writing – review & editing. **Yan Zhou:** Writing – original draft. **Albert J.R. Heck:** Writing – review & editing. **Yingrui Wang:** Writing – original draft. **Uwe Völker:** Writing – review & editing. **Xue Cai:** Writing – original draft. **Charles Boone:** Writing – review & editing. **Zizhuo Zhou:** Writing – original draft. **Ming Li:** Writing – review & editing. **Zhen Dong:** Writing – original draft. **Brenda Andrews:** Writing – review & editing. **Shuaiyao Wang:** Writing – original draft. **Joseph Schacherer:** Writing – review & editing. **Connie R. Jimenez:** Writing – review & editing. **Bernd Wollscheid:** Writing – review & editing. **Liang Yue:** Writing – original draft. **Ben C. Collins:** Writing – review & editing. **Heng Jiang:** Writing – original draft. **Dong Wang:** Writing – review & editing. **Liujia Qian:** Writing – original draft. **Jia-Xing Yue:** Writing – review & editing. **Yingying Sun:** Writing – original draft. **Edouard Nice:** Writing – review & editing. **Xiaofan Zhang:** Writing – original draft. **Yasset Perez-Riverol:** Writing – review & editing. **Jun A:** Writing – original draft. **Chris Sander:** Writing – review & editing. **Henning Hermjakob:** Writing – review & editing. **Wout Bittremieux:** Writing – review & editing. **Wilson Wen Bin Goh:** Writing – review & editing. **Ge Gao:** Writing – review & editing. **Peijie Zhou:** Writing – review & editing. **Siqi Sun:** Writing – review & editing. **Han Wen:** Writing – review & editing. **Ziyan Xu:** Writing – original draft. **Tiannan Guo:** Supervision, Writing – review & editing. All authors have read and approved the final manuscript.

## ORCID

0000-0002-8484-8089 (Rui Sun)

0000-0002-9576-3267 (Ruedi Aebersold)

0000-0002-6291-6722 (Qi Xiao)

0000-0003-1292-4799 (Matthias Mann)

0000-0003-3581-4701 (Yan Zhou)

0000-0002-2405-4404 (Albert J.R. Heck)

0000-0001-5532-3454 (Yingrui Wang)

0000-0002-5689-3448 (Uwe Völker)

0000-0002-7427-2100 (Xue Cai)

0000-0002-3542-6760 (Charles Boone)

0009-0002-7369-2573 (Zizhuo Zhou)

0000-0002-2157-2775 (Ming Li)

0000-0002-3480-3421 (Zhen Dong)

0000-0001-6427-6493 (Brenda Andrews)

0009-0001-1278-6958 (Shuaiyao Wang)

0000-0002-6606-6884 (Joseph Schacherer)

0000-0002-3103-4508 (Connie R. Jimenez)

0000-0002-3923-1610 (Bernd Wollscheid)

0000-0001-6478-7306 (Liang Yue)

0000-0003-0827-3495 (Ben C. Collins)

0009-0004-5281-4652 (Heng Jiang)

0000-0001-7877-7440 (Dong Wang)

0000-0002-0870-7479 (Liujia Qian)

0000-0002-2122-9221 (Jia-Xing Yue)

0009-0008-6015-2109 (Yingying Sun)

0000-0001-5480-4715 (Edouard Nice)

0009-0007-8777-6352 (Xiaofan Zhang)

0000-0001-6579-6941 (Yasset Perez-Riverol)

0009-0005-5524-8892 (Jun A)

0000-0001-6059-6270 (Chris Sander)

0000-0001-8479-0262 (Henning Hermjakob)

0000-0002-3105-1359 (Wout Bittremieux)

0000-0003-3863-7501 (Wilson Wen Bin Goh)

0000-0001-6470-8815 (Ge Gao)

0000-0002-4585-2923 (Peijie Zhou)

0000-0001-7240-8724 (Siqi Sun)

0000-0003-4680-4218 (Han Wen)

0000-0002-0678-8674 (Ziyan Xu)

0000-0003-3869-7651 (Tiannan Guo)
